# Reconstruction of Ovine Trachea with a Biomimetic Composite Biomaterial

**DOI:** 10.1155/2018/2610637

**Published:** 2018-10-17

**Authors:** Wojciech Ścierski, Grażyna Lisowska, Grzegorz Namysłowski, Maciej Misiołek, Jan Pilch, Elżbieta Menaszek, Radosław Gawlik, Marta Błażewicz

**Affiliations:** ^1^Department of Otorhinolaryngology and Laryngological Oncology, Medical University of Silesia, Zabrze, Poland; ^2^Department of Cytobiology, Collegium Medicum, Jagiellonian University, Krakow, Poland; ^3^Department of Biomaterials, AGH University of Science and Technology, Krakow, Poland; ^4^Department of Internal Medicine, Allergology and Clinical Immunology, Medical University of Silesia, Zabrze, Poland

## Abstract

The aim of this study was to evaluate a novel composite material for tracheal reconstruction in an ovine model. A polymer containing various forms of carbon fibers (roving, woven, and nonwoven fabric) impregnated with polysulfone (PSU) was used to create cylindrical tracheal implants, 3 cm in length and 2.5 cm in diameter. Each implant, reinforced with five rings made of PSU-impregnated carbon-fiber roving, had three external layers made of carbon-fiber woven fabric and the inner layer formed of carbon-fiber nonwoven fabric. The inner surface of five implants was additionally coated with polyurethane (PU), to promote migration of respiratory epithelium. The implants were used to repair tracheal defects (involving four tracheal rings) in 10 sheep (9-12 months of age; 40-50 kg body weight). Macroscopic and microscopic characteristics of the implants and tracheal anastomoses were examined 4 and 24 weeks after implantation. At the end of the follow-up period, outer surfaces of the implants were covered with the tissue which to various degree resembled histological structure of normal tracheal wall. In turn, inner surfaces of the prostheses were covered only with vascularized connective tissue. Inner polyurethane coating did not improve the outcomes of tracheal reconstruction and promoted excessive granulation, which contributed to moderate to severe stenosis at the tracheal anastomoses. The hereby presented preliminary findings constitute a valuable source of data for future research on a tracheal implant being optimally adjusted for medical needs.

## 1. Introduction

Reconstruction of tracheal defects >6 cm in length still constitutes a serious problem and represents a challenge for chest and neck surgeons. Tracheal defects may be associated with malignancy, mechanical injury, or stenosis caused by prolonged intubation or tracheotomy [1–3]. An optimal approach to tracheal repair is creation of a primary end-to-end anastomosis [4, 5]. However, application of this method is often limited by the defect's size; repair of defects that involve more than 50% of tracheal length (ca. 6 cm in adult patients) requires a reconstruction procedure [6].

In previous experimental and clinical studies, large tracheal defects were repaired with various materials, either autografts or synthetic implants, including pericardial or periosteal patches, costal, nasal septal or auricular cartilage, esophageal or urinary bladder wall, and many others [7–15]. However, such free flaps are not rigid enough to maintain the airway patency. The most satisfactory results were achieved with complex flaps composed of an alloplastic material conjugated with host tissues to form a single entity with shared vascular network [16–19]. Also some tissue engineering methods and allotransplantation techniques were tested for their potential application in tracheal reconstruction [20, 21]. However, these methods have some drawbacks and limitations as well, such as unavailability of tracheal prostheses in an emergency setting (management of acute posttraumatic defects), need for immunosuppressive therapy, ethical and legal issues, and granulation tissue formation [22, 23].

Large tracheal defects can be also reconstructed with synthetic materials. Initial experiments in this matter, most often involving canine trachea, included nonporous materials, such as stainless steel, vitallium alloy, tantalum, and many other metals. However, none of these studies produced satisfactory outcomes [14]. Aside from animal experiments, nonporous materials have been also sporadically used in a clinical setting [4]. However, researchers soon realized that markedly better outcomes of tracheal reconstruction can be obtained with porous materials. Due to the presence of pores, fibroblasts can easily migrate into the implant and form connective tissue layer on its inner surface; this provides better tightness of the prosthesis and better stability of tracheal anastomoses. Furthermore, connective tissue serves as a scaffold for respiratory epithelium migrating from adjacent tracheal segments. Most previous attempts to repair tracheal defects with porous materials involved synthetic meshes with various pore diameters, reinforced with wire spirals or plastic rings. Also nylon, Teflon®, Dacron®, and Marlex® meshes were used for tracheal reconstruction with variable outcomes. Principal challenges associated with the use of synthetic materials include restenosis resulting excessive growth of granulation tissue inside the implant, infections and resultant graft rejection, bleeding, and translocation of the prosthesis [19, 24–26].

However, the main problem that needs to be overcome during the reconstruction of large tracheal defects is stimulation of epithelial migration onto the whole inner surface of the implant. Lack of epithelial lining impairs transport of mucus and promotes granulation. Respiratory epithelium constitutes a barrier between host tissues and external environment, improving tightness of the trachea and protecting it against pathogens and foreign bodies penetrating from external environment. Furthermore, ciliated epithelial cells are involved in mucociliary clearance. Therefore, impaired and/or delayed migration of respiratory epithelium is associated with increased risk of infection and promotes formation of granulation tissue on the inner surface of the implant [27, 28]. Thus, synthetic material of the implant should show some activity to promote epithelial migration from adjacent tracheal segments. Another vital problem that needs to be solved are substantial differences in biomechanical properties of the trachea and synthetic prostheses.

The aim of this study was to evaluate a novel composite material for tracheal reconstruction in an ovine model. We used a polymer containing carbon fibers with various spatial architecture to obtain tracheal implants with anisotropic mechanical properties resembling characteristics of the ovine trachea.

## 2. Material and Methods

### 2.1. Biomechanical Study of the Ovine Trachea

**Figure 1:**
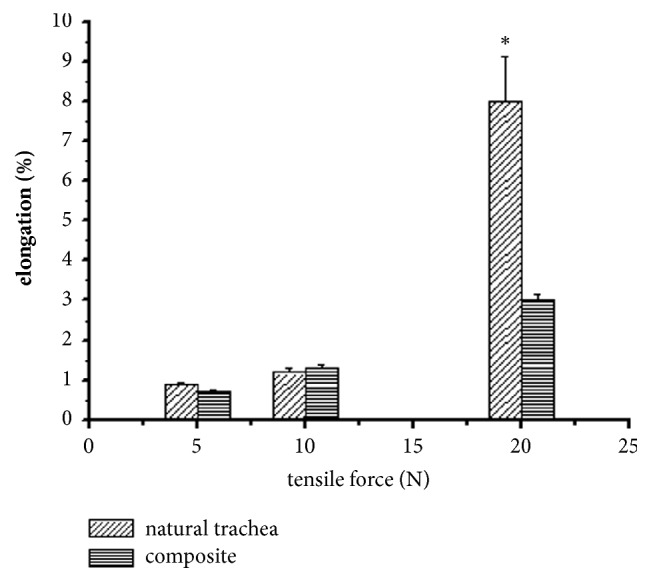
Comparison of mechanical properties of ovine trachea and composite implant under a tensile test. *∗*Statistically significant difference (p < 0.05).

The proper animal experiment was preceded by a biomechanical study of the ovine trachea. This was justified by the lack of published data documented anisotropic mechanical properties of this model. Specifically, we tested resistance of ovine tracheal tissues to stretching, bending, and squeezing with forces acting in various directions. Ovine trachea turned out to be an organ with anisotropic mechanical properties and deformability depending on the direction of applied force [29]. A review of published studies testing applicability of synthetic materials for tracheal reconstruction showed that all previously used implants (made of polymers, ceramic or metal) had isotropic properties. Therefore, their biomechanical characteristics differed considerably from those of the trachea. Based on the results of the previous biomechanical study of ovine trachea, we have designed a novel composite synthetic material containing carbon fibers and two types of polymers, i.e., polysulfone (PSU) and polyurethane (PU); biomechanical properties of this material closely resembled respective characteristics of ovine tracheal tissues. Before testing the novel composite biomaterial in animal experiment, its biomechanical properties were compared with the properties of natural ovine trachea. The specimens of ovine trachea and composite cylindrical implant were stretched at three levels of tensile force. As shown on Figure 1, both materials showed similar deformability under tensile forces corresponding to 5N and 10N, and the only differences were observed when higher tensile forces were applied. This implies that mechanical characteristics of the novel composite material resembled those of ovine trachea.

### 2.2. Preparation of Tracheal Implant

**Figure 2:**
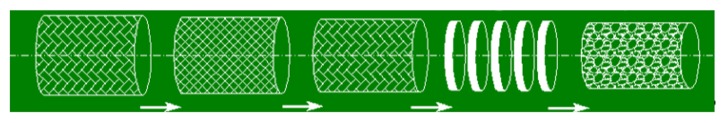
Schematic presentation of tracheal implant used in this study.

**Figure 3:**
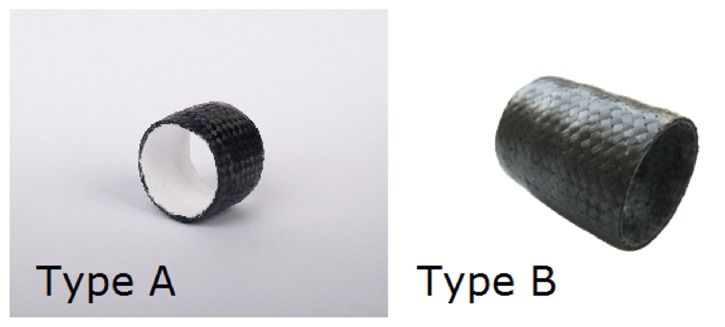
Two types of tracheal implants used in this study. (A) Implant with inner polyurethane coating. (B) Implant without the polyurethane coating.

A cylindrical tracheal implant, 3 cm in length and 2.5 cm in diameter, was designed. Its walls were made of the novel composite material containing various forms of carbon fibers (roving, woven and nonwoven fabric) impregnated with PSU. A scheme of the implant is presented on Figure 2. The implant was made of four composite layers in form of pipes, placed one over another. Three external layers were made of carbon-fiber woven fabric impregnated with PSU, whereas the fourth, inner layer was formed from PSU-impregnated carbon-fiber nonwoven fabric. The implant, in form of a pipe resembling the shape of ovine trachea, was reinforced with five rings made of carbon-fiber roving impregnated with PSU, similar to cartilaginous tracheal rings. In type A implants (n=5), the inner surface made of nonwoven fabric was additionally coated with PU, to promote migration of respiratory epithelium. Type B implants (n=5) were made solely of the PSU-impregnated composite material (Figure 3).

### 2.3. Animal Experiment

**Figure 4:**
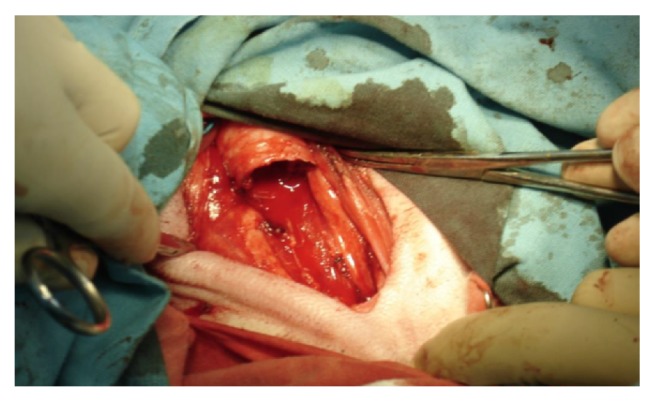
Resection of tracheal segment consisting of four rings.

**Figure 5:**
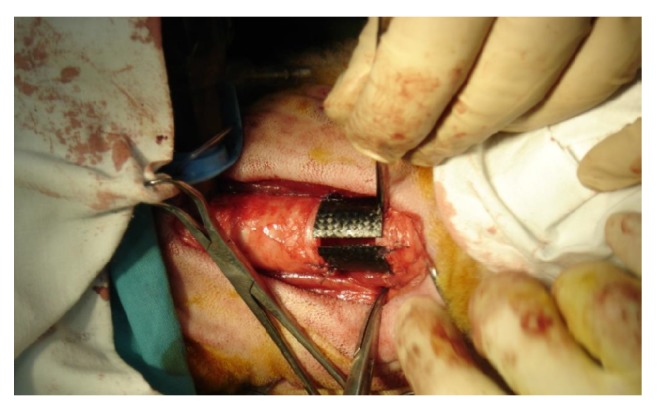
Reconstruction of tracheal defect with cylindrical implant.

The proper animal experiment included 9- to 12-month-old sheep with body weight of 40-50 kg. The protocol of the study was approved by the Local Bioethics Committee at the Medical University of Silesia in Katowice. The animals were divided into two groups (A and B, 5 animals each), implanted with different type A and type B tracheal prostheses, respectively. After premedication, each sheep was placed in a supine position. Upon circumferential mobilization of a 10-cm tracheal segment, a fragment consisting of four tracheal rings was removed (Figure 4). Resultant tracheal defect was repaired with the cylindrical implant fixed end-to-end using external 4-0 Prolene® sutures (Figure 5). Two animals from each group were euthanized 4 weeks after the procedure, and another three 24 weeks after surgery. Whole-length tracheal specimen containing the implant was cut of the larynx and main bronchi and removed. Then, transverse cuts were made 3 cm and 1 cm proximally from the upper tracheal anastomosis and 1 cm and 3 cm distally from the lower anastomosis. The degree of tracheal stenosis at the anastomosis site was estimated using a modified Hsieh's classification [30]. Tracheal lumen was considered unobstructed whenever the area of transverse cross-section at the anastomosis site corresponded to 75-100% of normal luminal area; reduction of luminal area at the anastomosis site down to 30-75% and <30% corresponded to moderate and severe tracheal stenosis, respectively.

**Figure 6:**
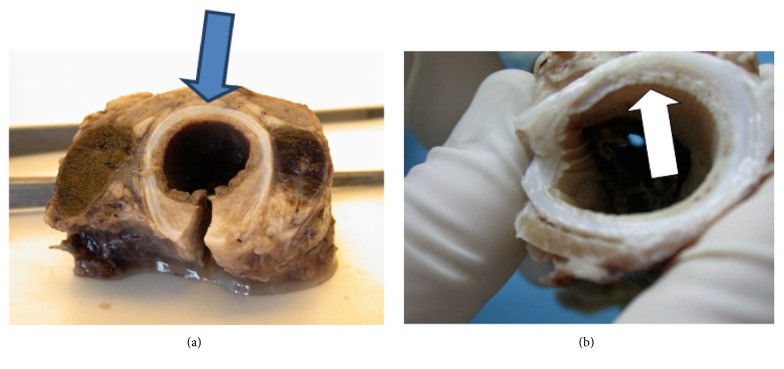
Topography of tissues subjected to microscopic analysis. (a) Tissues covering outer surface of the implant at the anastomosis site. (b) Tissues covering inner surface of the implant.

In most previous animal studies, histological analysis was limited to tissues covering inner surfaces of tracheal implants [22, 27, 28]. However, in this study, we examined both the tissues on the inner surface of the implant (van Gieson staining) and the tissues covering outer surface of the implant at the anastomosis site (Masson-Goldner staining and PAS staining) (Figure 6)

## 3. Results

Tracheal specimens from all animals from group A showed moderate (one sheep, euthanized 24 weeks after implantation) or severe stenosis at the anastomosis site. Apparently, high activity of PU-coated inner layer of type A implants stimulated excessive growth of granulation tissue, which underwent cicatrization, contributing to progressive stenosis at the anastomosis site.

Markedly better outcomes were observed in group B. Only one case of tracheal stenosis associated with excessive granulation at the anastomosis site was documented 4 weeks after implantation. No evidence of clinically relevant stenosis was found in the remaining four animals, including three sheep followed-up for 24 weeks (Table 1). Moreover, neither dehiscence at the anastomosis site nor damage/calcification of the tracheal rings was observed.

**Figure 7:**
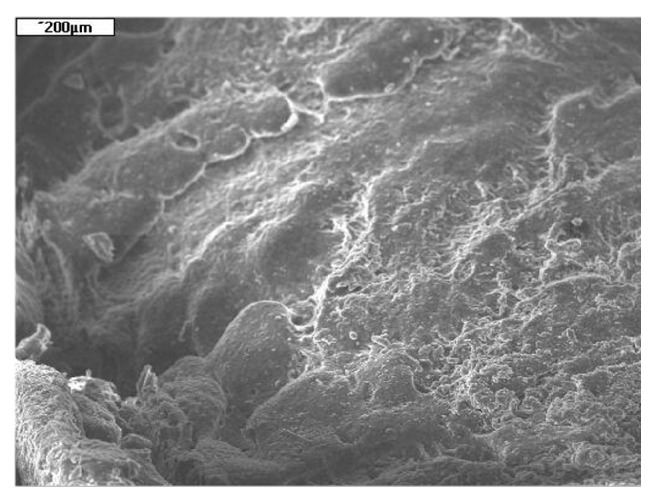
SEM microphotograph of tissue covering outer surface of the implant at the anastomosis site. Group A, 24 weeks after implantation. Tissues dried at 60°C for 24 h and coated with gold.

Prior to histological analysis, tracheal implants were separated from surrounding tissues. All implants seemed to be incorporated well by host trachea. The first stage of histological analysis was scanning electron microscopy (SEM). Microphotograph of tissues covering outer surface of the implant is shown on Figure 7.

**Figure 8:**
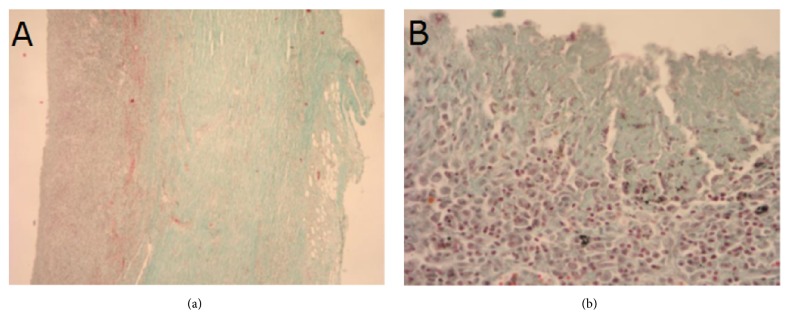
Histological structure of tissues covering inner surface of type A implant 4 weeks after implantation. (a) Lack of respiratory epithelium; instead a layer of connective tissue. (b) Numerous plasma cells; some cells contain particles of phagocyted carbon material. Lack of serous and mucinous glands. Masson-Goldner staining, ×4 (a) and ×40 (b).

**Figure 9:**
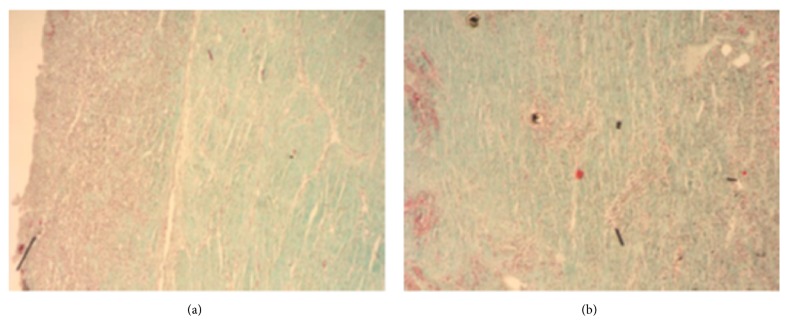
Histological structure of tissues covering inner surface of type B implant 4 weeks after implantation. Visible fragments of carbon fibers. Lack of mucinous glands in the area corresponding to submucosal tissues. Masson-Goldner staining, × 10.

**Figure 10:**
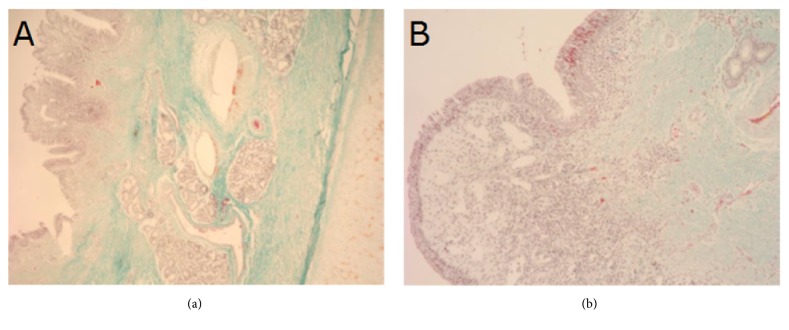
Histological structure of tissues covering outer surface of type A implant 24 weeks after implantation. Visible pseudostratified ciliated columnar epithelium, mucinous glands and cartilage. Masson-Goldner staining, ×4 (a) and ×10 (b).

**Figure 11:**
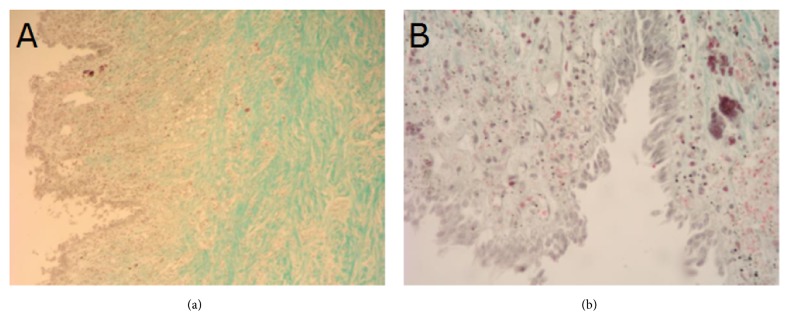
Histological structure of tissues covering outer surface of type B implant 24 weeks after implantation. Visible pseudostratified columnar epithelium covering tracheal wall. Carbon particles in both epithelial cells and cells of connective tissue stroma. Masson-Goldner staining, ×10 (a) and ×40 (b).

Histological structure of tissues adjacent to outer surfaces of type A and B implants at the anastomosis site 4 and 24 weeks after implantation are presented on Figures 8–11. As shown on the figures, microscopic structure of tissues covering the implants resembled that of normal tracheal wall to various degree (Figures 8–11).

**Figure 12:**
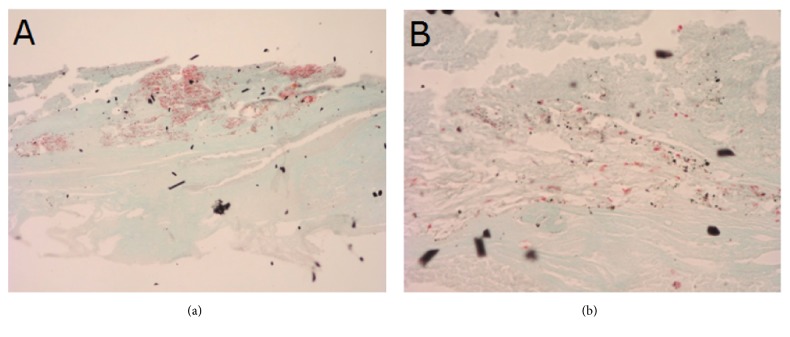
Histological structure of tissues covering inner surface of type B implant 24 weeks after implantation. Connective tissue composed primarily of collagen fibers. Visible blood vessels. Masson-Goldner staining, ×40 (a) and ×80 (b).

After 24 weeks of follow-up, inner surfaces of the implants were covered with patches of strongly bound connective tissue. Histological structure of connective tissue covering the inner surface of type B implant is presented on Figure 12. As shown on the figure, the tissue was primarily composed of collagen fibers, but also scattered newly formed blood vessels could be seen across the specimen.

## 4. Discussion

During recent several years, implants made of various synthetic materials have been tested for their applicability in tracheal reconstruction. However, none of them have found a wider application in reconstructive surgery since their biological and/or mechanical properties did not adequately mimic complex characteristics of tracheal tissues. Ideally, tracheal implant should be made of material that retains its shape, simultaneously being elastic and resistant to crushing and breaking. It should not be prone to deformation, for example, caused by high temperature or repeated strain. From a biological point of view, tracheal implants should no undergo rapid biodegradation, or their remodeling should be associated with penetration of connective tissue to micropores of the material. Inner surfaces of biomaterials used for tracheal reconstruction should show some biological activity to promote migration of pseudostratified ciliated epithelium and regeneration of airway mucosa [31]. Our previous studies demonstrated that ovine trachea represents a mechanical system with anisotropic properties [29]. None single synthetic material (whether based on a polymer, ceramic, carbon fibers or metal) has mechanical characteristics similar to biomechanical parameters of tracheal tissues. Therefore, we have designed a novel composite material with mechanical properties maximally resembling those of the ovine trachea. To this date, tracheal implants used in experimental studies were made of a single component (usually a polymer) and thus, lacked appropriate mechanical properties [14, 19, 32–35].

Schultz et al. [36, 37] tested cylindrical tracheal implants made of porous titanium (Ti40) in rat and ovine models. A total of 50% of rats and 30% of sheep survived till the end of a 3- to 12-month follow-up period. Tracheal specimens from these animals contained traces of a cylindrical ciliated respiratory epithelium on inner surfaces of the implants [36, 37]. Kaiser [34] used porous polyurethane patches to reconstruct small defects in the anterior wall of canine trachea. After 270 days of follow-up, Dacron® patches with 125-250 *μ*m pore size were incorporated well by the trachea in 6 out of 7 dogs, and their inner surfaces were covered with stratified ciliated epithelium [34]. Also Schauwecker et al. [38] observed good healing of larger circumferential tracheal defects (4 × 4 cm) repaired with polyurethane implants; however, tracheal lumen was obliterated with a newly formed granulation tissue. Also in our study, application of PU onto inner surface of type A implants resulted in excessive granulation tissue and progressive stenosis at the anastomosis site. In many previous animal experiments, tracheal defects were reconstructed with implants made of polytetrafluoroethylene (PTFE), a nonbiodegradable polymer used widely in reconstructive vascular surgery. Tracheal implants made of PTFE were usually reinforced with outer spiral rings resembling tracheal rings. Guijarro Jorge et al. [33] used such prostheses, reinforced with spiral silicone rings, to repair 4-cm circumferential tracheal defects in 10 rabbits. Tracheal specimens from 5 rabbits that have survived till the end of a 6-month follow-up period showed high degree of epithelialization on inner surfaces of the implants; overall, complete epithelialization of the implants was documented in 7 out of 10 animals. According to the authors of this study, successful outcome of tracheal reconstruction might have been inter alia associated with adequate size of pores in the implanted material (30 *μ*m), enabling penetration of blood vessels from surrounding tissues onto the inner surface of the prosthesis [33]. Slightly less favorable experiences with PTFE implants were reported by Shaha et al. [35] who used this material to repair tracheal defects in 26 dogs. Histological examination of revealed considerable fibrosis around all graft; respiratory epithelium was found solely on the inner surfaces of the implants adjacent to tracheal anastomoses [35]. Even less favorable outcomes of tracheal reconstruction with PTFE implants were reported by Cull et al. [39] who used this material to repair 5-cm tracheal defects in nine dogs. Postmortem examination showed no evidence of epithelialization on inner surfaces of the implants that were surrounded by large deposits of fibrous connective tissue [39]. As shown above, the results of previous studies dealing with tracheal implants made of PTFE are quite inconclusive, both with regard to formation of granulation tissue at the anastomosis site and in terms of migration of respiratory epithelium onto inner surfaces of the prostheses. While PTFE is not rigid enough to prevent the airway collapse, it seems to promote migration of respiratory epithelium better than the composite carbon material used in our present study.

Okumura et al. [40] used 3-cm cylindrical implants made of Marlex® mesh reinforced with spiral polypropylene rings. To obtain better airtightness and to promote connective tissue infiltration, inner surfaces of the implants were coated with collagen from porcine skin. The authors performed segmental tracheal resection in 13 dogs. Postmortem examination of five animals that survived at least 6 months after the reconstruction showed complete incorporation of the prosthesis by the host trachea along with confluent epithelialization of its inner surface with respiratory epithelium [40].

Although duration of follow-up in previous animal experiments varied, according to most authors, 24 weeks is sufficiently long period to achieve complete healing of tracheal anastomosis. Previous studies demonstrated that epithelial healing starts as early as 7-14 days after surgery; however, the epithelium covering tracheal anastomosis has no cilia, is composed of less layers, and shows the signs of metaplasia [41, 42]. Similar to other authors, we have followed-up the study animals for 24 weeks. None of the histological specimens showed presence of respiratory epithelium across the whole inner surface of the tracheal implants. However, inner surfaces of all implants were covered with patches of strongly bound connective tissue containing newly formed blood vessels.

## 5. Conclusions

In this study, tracheal defects were repaired with implants made of a composite material containing three types of PSU-coated carbon fibers (roving, woven and nonwoven fabric). Carbon fibers provided appropriate biomimetic characteristics of the implant, such as anisotropy, elasticity, and durability. Inner surfaces of some implants were additionally coated with polyurethane. At the end of the follow-up period, outer surfaces of the implants were covered with the tissue which to various degree resembled histological structure of normal tracheal wall. However, inner surfaces of the prostheses were covered only with vascularized connective tissue. Inner polyurethane coating did not improve the outcomes of tracheal reconstruction. The hereby presented preliminary findings constitute a valuable source of data for future research on a tracheal implant being optimally adjusted for medical needs.

## Figures and Tables

**Table 1:** Degree of stenosis at the tracheal anastomosis, according to Hsieh's classification.

	Mild stenosis	Moderate stenosis	Severe stenosis
Group A	0	1	4
Group B	4	1	0

## Data Availability

The data used to support the findings of this study are available from the corresponding author upon request.

## Abbreviations

PSU: Polysulfone
PU: Polyurethane
SEM: Scanning electron microscopy
PTFE: Polytetrafluoroethylene.
